# Spiral artery blood flow during pregnancy: a systematic review and meta-analysis

**DOI:** 10.1186/s12884-020-03150-0

**Published:** 2020-11-11

**Authors:** Veronique Schiffer, Laura Evers, Sander de Haas, Chahinda Ghossein-Doha, Salwan Al-Nasiry, Marc Spaanderman

**Affiliations:** 1 Department of Obstetrics and Gynecology, Maastricht University Medical Centre (MUMC+), P. Debeyelaan 25, 6229 HX Maastricht, The Netherlands; 2 GROW School for Oncology and Developmental Biology, Maastricht University, Maastricht, The Netherlands; 3 Department of Cardiology, Maastricht University Medical Centre (MUMC+), Maastricht, The Netherlands

**Keywords:** Spiral artery, Doppler, Pregnancy, Adaptation, Remodeling

## Abstract

**Background:**

Downstream remodeling of the spiral arteries (SpA) decreases utero-placental resistance drastically, allowing sustained and increased blood flow to the placenta under all circumstances. We systematically evaluated available reports to visualize adaptation of spiral arteries throughout pregnancy by ultra-sonographic measurements and evaluated when this process is completed.

**Methods:**

A systematic review and meta-analysis of spiral artery flow (pulsatility index (PI), resistance index (RI) and peak systolic velocity (PSV)) was performed. English written articles were obtained from Pubmed, EMBASE and Cochrane Library and included articles were assessed on quality and risk of bias. Weighted means of Doppler indices were calculated using a random-effects model.

**Results:**

In healthy pregnancies, PI and RI decreased from 0.80 (95% CI: 0.70–0.89) and 0.50 (95% CI: 0.47–0.54) in the first trimester to 0.50 (95% CI: 0.45–0.55, *p* < 0.001) and 0.39 (95% CI: 0.37–0.42, *p* < 0.001) in the second trimester and to 0.49 (95% CI: 0.44–0.53, *p* = 0.752) and 0.36 (95% CI: 0.35–0.38, *p* = 0.037) in the third trimester, respectively. In parallel, PSV altered from 0.22 m/s (95% CI: 0.13–0.30 m/s) to 0.28 m/s (95% CI: 0.17–0.40 m/s, *p* = 0.377) and to 0.25 m/s (95% CI: 0.20–0.30 m/s, *p* = 0.560) in the three trimesters. In absence of second and third trimester Doppler data in complicated gestation, only a difference in PI was observed between complicated and healthy pregnancies during the first trimester (1.49 vs 0.80, *p* < 0.001). Although individual studies have identified differences in PI between SpA located in the central part of the placental bed versus those located at its periphery, this meta-analysis could not confirm this (*p* = 0.349).

**Conclusions:**

This review and meta-analysis concludes that an observed decrease of SpA PI and RI from the first towards the second trimester parallels the physiological trophoblast invasion converting SpA during early gestation, a process completed in the midst of the second trimester. Higher PI was found in SpA of complicated pregnancies compared to healthy pregnancies, possibly reflecting suboptimal utero-placental circulation. Longitudinal studies examining comprehensively the predictive value of spiral artery Doppler for complicated pregnancies are yet to be carried out.

## Background

The most important functions of the placenta are adequate exchange of oxygen, nutrients and waste products between fetal and maternal circulation [1]. At 12–13 weeks of gestation, trophoblast cells have invaded the decidual segments of maternal spiral arteries (SpA) and transformed these small, adrenergic-sensitive high-resistance vessels into wide, adrenergic-insensitive low-resistance vessels [1, 2]. From 15 weeks onwards, a second, endovascular invasion starts to remodel the myometrial segments and is fully completed at mid-pregnancy [3]. Downstream remodeling of the SpA decreases utero-placental resistance drastically and allows significant increase in volumetric blood flow to the placenta [1, 4–7]. Interestingly, there are studies showing that trophoblast invasion is concentrated in the central area of the placental bed, whereas peripheral myometrial segments are much less changed [8, 9].

The etiology of placenta syndrome (PS), a collective term for placenta-insufficiency related disorders, is poorly understood. Inadequate remodeling of SpA in PS-pregnancies potentially affects the development of the placenta as high velocity blood flow could cause mechanical stress on the tissue, or could influence development due to perfusion-reperfusion injury [6, 7, 10].

The golden standard for the detection of absent or aberrant SpA remodeling is postpartum histopathological examination of the placenta, however this is behind time to affect clinical decision making in endangered gestation [10]. Previous studies have shown that abnormal Doppler velocimetry of the more upstream uterine arteries in second trimester is associated with an increased risk on vascular pregnancy related complications (among which pre-eclampsia (PE), fetal growth restriction (FGR) and stillbirth) [11, 12]. Abnormal uterine artery Doppler indices during earlier gestation may also predict PE and FGR [13, 14]. As defective SpA remodeling ultimately associates with adverse outcome, capturing this incomplete SpA remodeling antedating complicated pregnancies during early gestation may timely indicate those at risk [1, 15]. Our current knowledge on SpA remodeling is mostly based on previous literature discussing postpartum histological findings and cross-sectional ultrasound measurements. However, longitudinal ultrasound data throughout pregnancy would provide a more accurate insight in the SpA remodeling process during gestation. Therefore, we performed a systematic review and meta-analysis on Doppler measurements of the SpA, to antepartum visualize the remodeling process in human pregnancy by ultrasonography.

## Methods

This systematic review and meta-analysis on the remodeling of SpA flow velocimetry throughout human pregnancy is part of a large series of meta-analysis on physiological and pathophysiological adaptation of relevant indices in pregnancy [16–18]. The review was conducted in accordance to the “PRISMA Statement” for reporting systematic reviews and meta-analyses [19].

### Literature search

The electronic databases Cochrane library (1997–2019), Pubmed (1946–2019) and EMBASE (1974–2019), were searched for relevant articles evaluating SpA Doppler measurements. The keywords used in the literature search were “spiral artery” or “spiral remodeling” in combination with “Doppler” or “color Doppler”, or “Doppler velocimetry”, or “Doppler sonography” or “spiral artery Doppler”, see Table 1.

**Table 1:** Search strategy (Pubmed database)

For the electronic search of Cochrane Library and Embase we used a more broad search using a combination of terms ‘spiral artery’ AND ‘Doppler’ (no limits or filters)	
Search (spiral artery) AND Doppler Search Pubmed (no limits or filters) 1. Spiral artery [All Fields] 2. Spiral artery remodeling [All Fields] 3. Search 1 OR 2 4. Color Doppler ultrasonography [Mesh] 5. Doppler [All Fields] 6. Doppler sonography [All Fields] 7. Doppler velocimetry [All Fields] 8. Pulse wave Doppler [All Fields] 9. Search 4 OR 5 OR 6 OR 7 OR 8 10. Search 3 AND 9	

Additionally, the reference list of all selected primary articles were examined for potential citations not captured by the initial search. The search was limited to papers published in English until April 2019.

### Study selection

The first selection, based on title and abstract, was performed independently by two investigators (VS and LE). In case of discrepancy, agreement was reached by consensus. The second selection was also performed by two investigators (VS and LE) independently, based on the full text. After checking the manuscripts and crosschecking their reference lists, the final selection of studies was made. Inclusion criteria were studies on singleton pregnancies in which absolute values for SpA Doppler measurements were documented at any gestational age during pregnancy. Studies with both nulliparous and multiparous women were included; no limitations were set on maternal characteristics. Moreover, both transvaginal and transabdominal measurements were included as well as measurements of central localized and peripheral localized SpA. We initially wanted to include only articles describing SpA measurements in healthy and hypertensive pregnancies. Given the limited amount of articles describing SpA measurements in hypertensive pregnancies, we decided to include all articles describing SpA measurements in complicated pregnancies. Therefore, complicated pregnancies in the included articles were represented by PE, preterm labor, FGR, missed abortion, miscarriage and placental abruption.

In some studies, absolute values of Doppler indices could not be extracted as a result of data being reported in graphs or figures. We considered the use of, for example, a plot digitizer to extract the data out of graphs or plots. The accuracy of the assessment was in some cases questionable, and in other cases, not all graphs and plots showed 95% confidence interval (CI) lines, making it impossible to calculate a reliable standard deviation (SD). Therefore, adding a plot digitized estimated SpA value would have a great impact on the trustworthiness of the pooled mean. Therefore, we refrained from generating extra data using the plot digitizer as it may be less accurate compared to the other included articles.

### Data extraction and quality assessment

Two reviewers (VS and LE) both extracted the data and assessed the risk of bias. In case of discrepancy in assessment of the risk of bias, agreement was reached by consensus. The following data was extracted from each included study: 1) Study design; 2) Sample size; 3) Probe; 4) Doppler method 5) MHz of the probe; 6) Gestational age during measurement; and 7) Doppler velocimetry outcomes (Table 2). The relevant outcomes included pulsatility index (PI), resistance index (RI) and peak systolic velocity (PSV, m/s).

**Table 2:** Baseline characteristics from included studies

	Study design	Healthy pregnancies (n)	Complicated pregnancies (n)	Probe	Doppler	MHz	Gestational age at measurement (weeks)	Outcome
**Alouini** (**ref**. [20])	Prospective longitudinal	49	/	Transvaginal	Colour	6	5–10	PSV
**Coppens **(**ref**. [21])	Prospective longitudinal	37	/	Transvaginal	Colour	6–9	8–14	PI
**Deurloo** (**ref**. [2])	Prospective longitudinal	86	21^a^	Transabdominal	Colour	3.5	11–13; 14–17; 18–24	PI, RI
**Deurloo **(**ref**. [22])	Prospective longitudinal	97	/	Transabdominal	Colour	3.5	11–13; 14–17; 18–24	PI
**Hsieh** (ref. [23])	Prospective longitudinal	94	/	Transabdominal	Colour	3.5	10–15; 15–20; 20–25; 25–30; 30–35; 35–40	PI, RI, PSV
**Kurjak** (**ref**. [24])	Cross-sectional	115	/	Transvaginal + transabdominal	Colour	3.5 / 5	7–42	PI, RI, PSV
**Kurjak** (**ref**. [25])	Prospective cross-sectional	60	54^a^	Transvaginal	Colour	6	6–12	PI, RI
**Mäkikallio, Jouppila** (**ref**. [26])	Prospective cross-sectional	31	10^c^	Transvaginal	Colour	5	6; 8; 9; 11	RI
**Mäkikallio, Tekay** (**ref**. [27])	Prospective longitudinal	16	/	Transabdominal	Colour	5	5; 7; 8; 10	PI, PSV
**Matijevec** (**ref**. [9])	Prospective cross-sectional	64	/	Transabdominal	Colour	5	17–20	PI, RI
**Ozkan** (**ref**. [15])	Prospective longitudinal	189	25^b^	Transvaginal	Colour	12	5–12	PI, RI
**Ozkaya** (**ref**. [28])	Prospective cross-sectional	84	16^c^	Transvaginal	Colour	6	6–12	PI, RI

*N* amount patients, *MHz* Megahertz, *PI* pulsatility index, *RI* resistance index, *PSV* peak systolic velocity

^a^Absolute data not available

^b^Miscarriage

^c^Pre-eclampsia, preterm labour, intra uterine growth restriction, missed abortion, miscarriage and placental abruption

In order to assess the quality and risk of bias of included studies, the Quality In Prognosis Studies (QUIPS) tool [29] was modified for the purpose of this review. Quality assessment was performed on the following domains: 1) Study participation; 2) Study attrition; 3) Outcome measurement; 4) Data reporting; and 5) Study design (Table 3).

**Table 3:** Quality assessment of included studies

Domain	Items for consideration	Alouini (ref. [20])	Coppens (ref. [21])	Deurloo (ref. [2])	Deurloo (ref. [22])	Hsieh (ref. [23])	Kurjak (ref. [24])	Kurjak (ref. [25])	Mäkikallio (ref. [26])	Mäkikallio (ref. [27])	Matijevic (ref. [9])	Ozkan (ref. [15])	Ozkaya (ref. [28])
**Study participation**	Adequate description of participants’ characteristics												
	• Parity or gravidity	–	–	–	+	+	–	–	+	–	–	–	+
	• Health or comorbidities of participants	–	–	+	+	+	–	+	+	–	–	–	–
	• Clear reporting of weeks amenorrhea	+	+	+	+	+	+	+	+	+	+	+	+
	• Ethnicity	–	–	–	–	–	–	–	–	–	–	–	–
	• Age	–	–	+	+	+	–	+	+	+	+	+	+
	• Non-pregnant weight/BMI	–	–	+	+	–	–	–	–	–	–	–	–
	• Use of medication or supplements	–	–	–	–	–	–	+	–	–	–	–	–
	Adequate description of participant recruitment	–	–	+	+	+	–	+	–	+	+	+	+
	Adequate description of inclusion and exclusion criteria	–	–	+	+	+	+	+	+	+	–	+	+
**Study attrition**	Reasons for loss to follow-up/drop-out are provided	–	+	–	–	–	–	–	–	–	–	–	–
	Adequate description of participants lost to follow-up/ differences between participants who completed and drop-outs	–	–	–	–	–	–	–	–	–	–	–	–
**Outcome measurement**	Method of measurement is adequately valid and reliable	+	+	+	+	+	+	+	+	+	+	+	+
	The methods and setting are the same for all study participants and throughout follow up	+	+	+	+	+	–	+	+	+	+	+	+
**Data reporting**	Data is extractable as mean with standard deviation for analysis	+	+	+	+	+	+	+	+	+	+	+	+
**Study design**	Study used a longitudinal study design	–	+	+	+	+	+	–	+	+	–	–	–
	Multiple (> 2) longitudinal pregnant measurements during pregnancy of the variable	?	+	+	+	+	+	?	+	+	?	?	?
	Assessment of intra- and/or inter-observer variation is performed	–	–	+	+	–	–	–	+	+	–	–	–
	**Count (+)**	4	7	12	13	11	5	9	11	10	6	7	8
	**Score %**	24	41	71	76	65	29	53	65	59	35	41	47
	**Quality**	**Low**	**Medium**	**High**	**High**	**High**	**Low**	**Medium**	**High**	**Medium**	**Low**	**Medium**	**Medium**

*Ref.* reference number

Scoring of each criteria occurred as insufficient [−] or sufficient [+]. In case an item was not applicable for the study, a question mark [?] was used. Based on the number of [+], the total score as a percentage was calculated. Articles scoring < 30% were defined as low quality, between 30 and 60% as moderate quality, and > 60% as high quality.

### Statistical analysis

The obtained SpA measurements were categorized into three pregnancy trimesters and divided by healthy versus complicated pregnancies and central versus peripheral SpA. Some articles performed two or more SpA measurements in one pregnancy trimester in the same population, explaining the repeated presence of one article in the forest plots. The analysis was conducted by using the R Project for Statistical, R version 3.4.0 with ‘meta’package version 2.0–0 [30]. In few instances, the continuous outcome measures were not presented with their corresponding SD but as a standard error or 95% CI. In these cases, a SD was calculated from the available mean and range according to the Cochrane handbook for Systematic Review of Interventions [31]. SpA weighted means with 95% CI were calculated separately for the predefined trimesters using a random-effects model, as described by DerSimonian and Laird [32]. This model allows for inter-study variation and was chosen because heterogenic populations (both healthy and complicated pregnancies) were used. Means and standard deviations were pooled into one combined measurements for studies reporting multiple measurements within a pregnancy trimester. Heterogeneity was explored as the ratio between total heterogeneity and total variability with the I^2^ statistic. I^2^ can differentiate between true heterogeneity and sampling variance [33]. Differences in SpA measurements between trimesters were considered statistically significant at *p* < 0.05.

## Results

### Baseline features

**Fig. 1:**
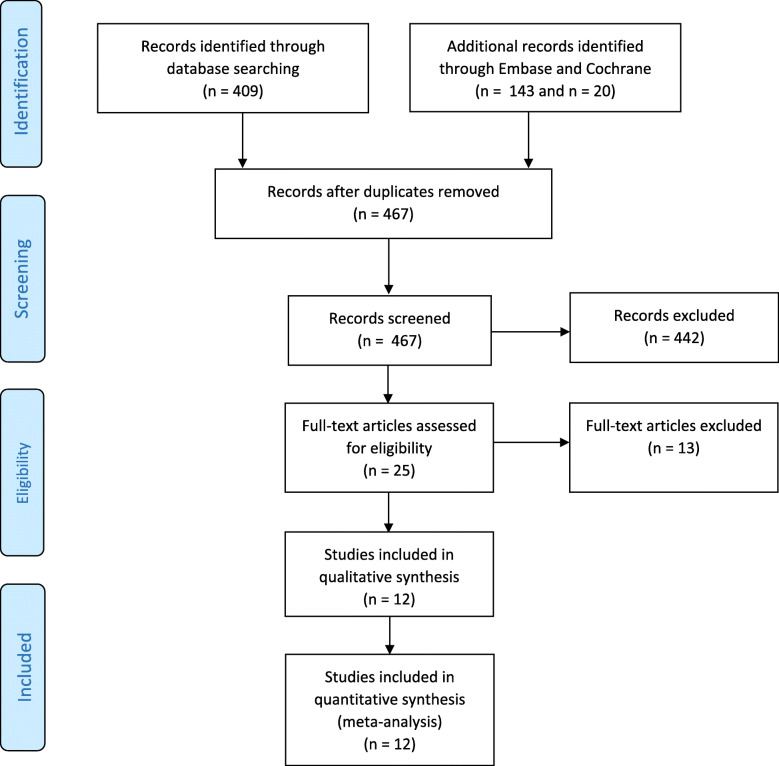
PRISMA 2009 flow diagram. *N* = amount of articles

Figure 1 graphically illustrates the flowchart of the article selection. A total of 559 records were identified by the search in Pubmed (*n* = 409), Embase (*n* = 143) and Cochrane (*n* = 20). No restrictions were set on publication status. We were unable to add any extra articles that were not identified by the literature search after examination of the reference lists. After removal of duplicates (*n* = 92), 467 articles were screened for eligibility based on title and abstract. Of these, 442 records were discarded based on title and abstract because these papers investigated other study outcomes, included animal studies, were reviews or were written in a non-English language that the investigators did not understand. Of the remaining 25 records, full-text articles were retrieved and examined in detail for eligibility. Thirteen additional studies were excluded, due to only estimates in figures were presented of spiral artery Doppler indices and no absolute values. We contacted the authors of whom their correct contact information was reported in the articles, to see whether they were able to provide additional data. Unfortunately, only two authors replied that they could not help us, since the research was performed before digitalization and it was not possible to acquire the absolute data of the dated investigations.

A total of 12 studies published in English met the eligibility criteria and were included in this systematic review and meta-analysis. The selected articles were longitudinal prospective (*n* = 7) and cross-sectional studies (*n* = 5). SpA measurements were obtained using transvaginal (*n* = 6), transabdominal ultrasound (*n* = 5) or both (*n* = 1) with probes between 3.5 to 12 MHz. All articles evaluated SpA Doppler during healthy pregnancy. Besides, five articles investigated the differences in SpA Doppler measurements between complicated and healthy pregnancies. There were no articles found that examined SpA Doppler measurements in complicated pregnancies only. Detailed information on the characteristics of included studies is presented in Table 2.

### Quality assessment of the included studies

The quality assessment according the modified QUIPS tool per domain is summarized in Table 3. Along these lines, most articles were classified as moderate quality study (*n* = 5) [15, 21, 25, 27, 28]. Alouini et al. [20], Kurjak et al. [24] and Matijevec et al. [9] were scored low quality, whereas Mäkkikallio, Tekay et al. [26], Hsieh et al. [23] and the two articles of Deurloo et al. [2, 22] scored high quality (Table 3). Ethnicity, non-pregnant BMI and use of medication were poorly described in most studies. In addition, 11 of the 12 included studies failed to report lost to follow up. Intra- and/or inter-observer variation was reported scarcely (*n* = 4).

### Meta-analysis

Studies reported Doppler indices at different gestational ages. If one mean SpA Doppler parameter was reported with an interval of gestational weeks as time of measurement, we included the mean of the interval as time of measurement in the meta-analysis (e.g. measurement performed between 6 and 12 weeks; mean gestational age at measurement 9 weeks). Doppler indices were combined based on measurements during first trimester (0–14 weeks), second trimester (15–27 weeks) or third trimester (28–40 weeks).

#### Pulsatility index

**Fig. 2:**
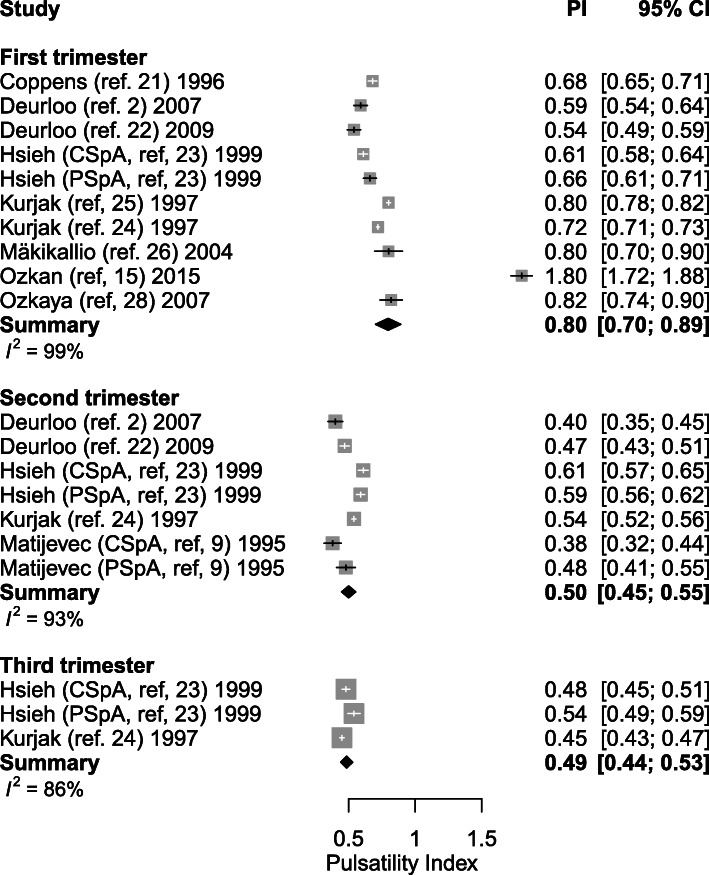
Forest plot of studies exploring spiral artery measurements (Pulsatility Index) during first, second and third trimester in healthy pregnancies

**Fig. 3:**
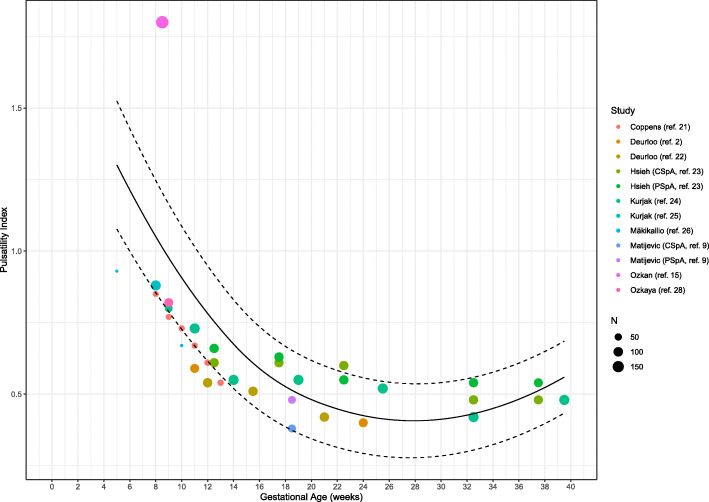
Meta-regression curve of spiral artery measurements (Pulsatility Index) during first, second and third trimester in healthy pregnancies. Every color is representing one article, except for Matijevic et al. (ref. [9]) and Hsieh et al. (ref. [23]) who have two colors representing central and peripheral located spiral artery measurements. ‘N’ represents the maximum amount of cases included in the study, shown as a small or larger circle

Ten studies explored the PI at certain (between 5 and 39.5 weeks of gestation) time-points during healthy gestation. The weighted mean of PI decreased from 0.80 (95% CI: 0.70–0.89) (*n* = 9) in the first trimester to 0.50 (95% CI: 0.45–0.55, *p* < 0.001) (*n* = 5) in the second trimester and to 0.49 (95% CI: 0.44–0.53, *P* = 0.752) (*n* = 2) in the third trimester (Table 4, Figs. 2 and 3). One thousand three hundred nine SpA were measured during the first trimester in 778 women. Five studies performed assessment during the second trimester, capturing 1011 SpA measurements in 456 women [2, 9, 22–24]. Two studies published 593 SpA Doppler PI measurements during the third trimester measured in 209 women [23, 24].

**Table 4:**
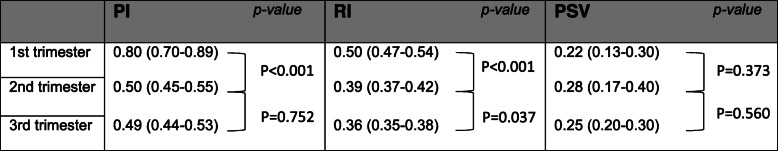
*P*-values showing significance level between weighted means of first, second and third trimester PI, RI and PSV in healthy pregnancies

#### Resistance index

**Fig. 4:**
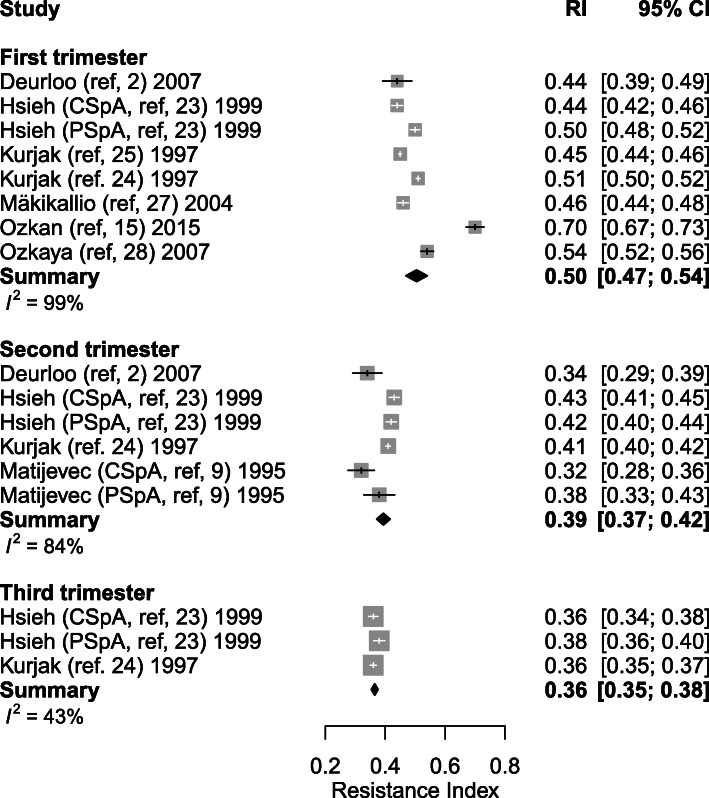
Forest plot of studies exploring spiral artery measurements (Resistance Index) during first, second and third trimester in healthy pregnancies

**Fig. 5:**
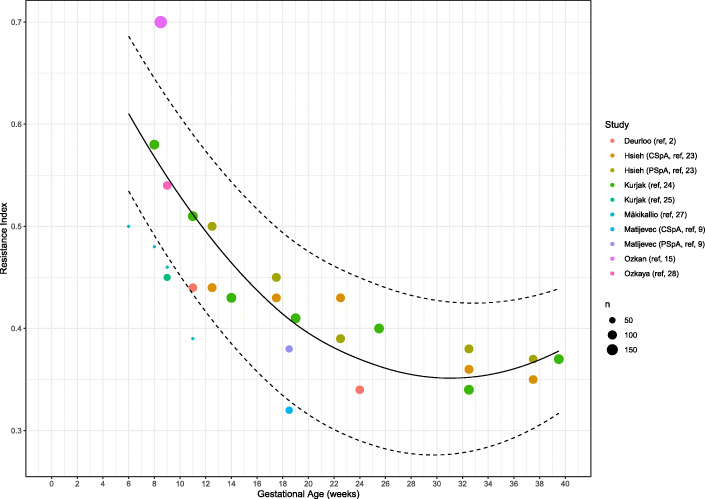
Meta-regression curve of spiral artery measurements (Resistance Index) during first, second and third trimester in healthy pregnancies. Every color is representing one article, except for Matijevic et al. (ref. [9]) and Hsieh et al. (ref. [23]) who have two colors representing central and peripheral located spiral artery measurements. ‘N’ represents the maximum amount of cases included in the study, shown as a small or larger circle

Eight studies explored the RI at different time-points during healthy pregnancy (between 6 and 39.5 weeks of gestation). The weighted mean of RI showed a decrease from 0.50 (95% CI: 0.47–0.54) (*n* = 7) in the first trimester to 0.39 (95% CI: 0.37–0.42, *p* < 0.001) (*n* = 4) in the second trimester and 0.36 (95% CI: 0.35–0.38, *p* = 0.037) (*n* = 2) in the third trimester (Table 4, Figs. 4 and 5). Only four studies performed measurements during the second [23–26] and two studies during the third trimester [23, 24]. One thousand and seventy-six SpA were measured during the first trimester in 659 women. A total of 820 SpA were measured in 359 women during second trimester and 593 SpA were measured in 209 women during third trimester.

#### Peak systolic velocity

**Fig. 6:**
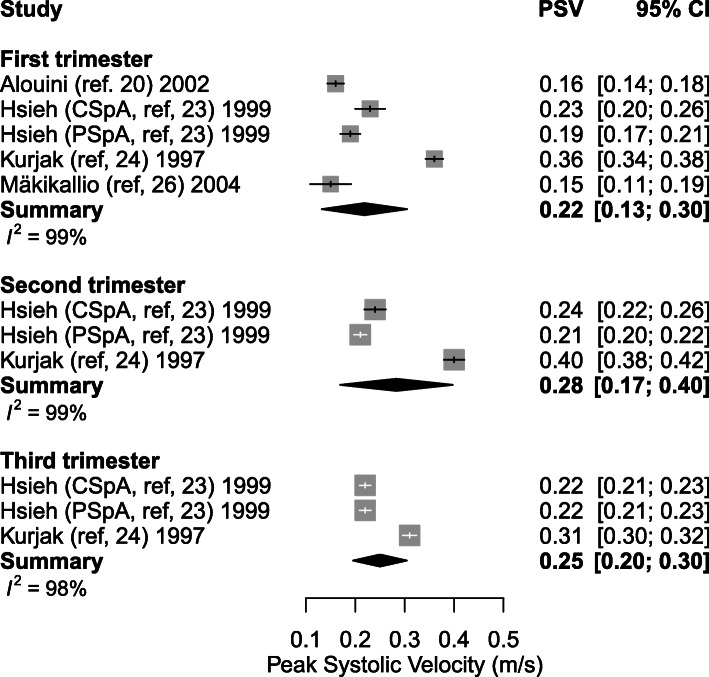
Forest plot of studies exploring spiral artery measurements (Peak Systolic Velocity m/s) during first, second and third trimester in healthy pregnancies

**Fig. 7:**
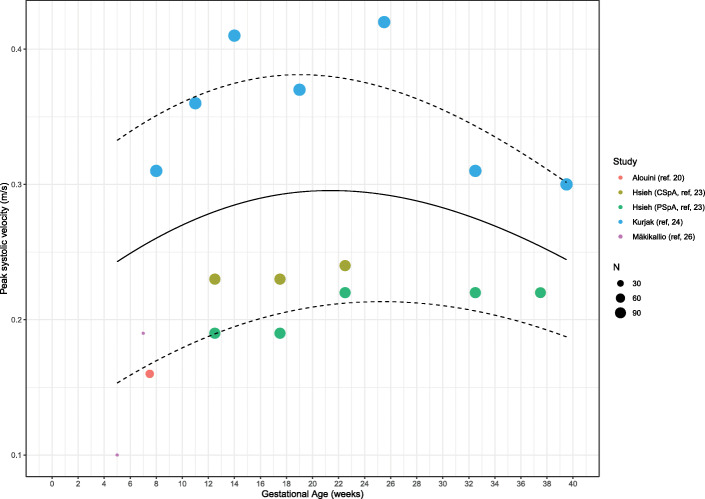
Meta-regression curve of spiral artery measurements (Peak Systolic Velocity) during first, second and third trimester in healthy pregnancies. Every color is representing one article, except for Hsieh et al. (ref. [23]) who have two colors representing central and peripheral located spiral artery measurements. ‘N’ represents the maximum amount of cases included in the study, shown as a small or larger circle

PSV during the first trimester of healthy pregnancy was explored in four individual studies (between 5 and 39.5 weeks of gestation). The weighted mean of PSV increased from 0.22 m/s (95% CI: 0.13–0.30) (*n* = 3) in the first trimester to 0.28 m/s (95% CI: 0.217–0.40, *p* = 0.373) (*n* = 2) in the second trimester and remained 0.25 m/s (95% CI: 0.20–0.30, *p* = 0.560) (*n* = 2) in the third trimester (Table 4, Figs. 6 and 7). During the first trimester, 614 SpA were measured in 274 women. Hsieh et al. [23] together with Kurjak et al. [24] performed measurements in the second and third trimester, including a total of 209 women and 606, respectively 593 SpA measurements.

#### Complicated pregnancies

**Fig. 8:**
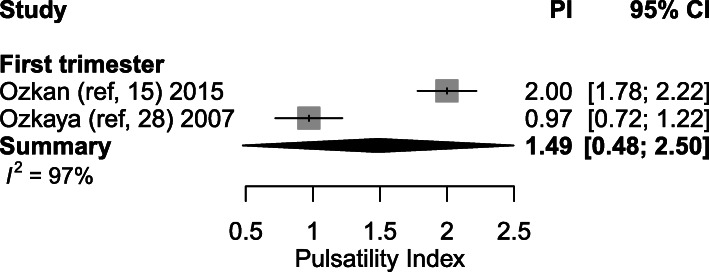
Forest plot of 2 studies exploring spiral artery measurements (Pulsatility Index) during first trimester in complicated pregnancies

Four articles [2, 15, 25, 28] investigated SpA PI during first trimester in complicated pregnancies (including PE, preterm labour, FGR, pregnancy induced hypertension, anembryonic pregnancies, missed abortion, miscarriage and placental abruption). However, absolute mean PI measurements were only described in two articles during the first trimester [15, 28]. Non-significant higher SpA PI measurements were found by Ozkan et al. in 25 women with subsequent miscarriages compared to 189 women having continuing subsequent pregnancies (2.0 (95% CI: 1.6–2.7) vs 1.8 (95% CI: 1.1–2.9), *p* = 0.320). Results of Ozkaya et al. showed mean SpA PI in 16 pregnancies with adverse outcome (3 miscarriage, 6 missed abortion, 2 preterm labour, 3 IUGR, 1 PE, 1 placental abruption) was 0.97 ± 0.51 (mean ± SD) vs 0.82 ± 0.39 in 84 women with normal pregnancy outcome. The difference in PI between both groups was non-significant. When pooling these data, a statistically significant difference between complicated and healthy pregnancies in the first trimester was found (1.49 vs 0.80, *p* < 0.001, Fig. 8 vs Fig. 3). No data was available on PI measurements in second or third trimester pregnancies.

Both articles, along with a third article [27], investigated SpA RI during the first trimester in a total of 51 women. Non-significant differences in RI were found by Ozkan et al. between women with subsequent miscarriages compared to women having continuing subsequent pregnancies (0.7 (95% CI: 0.6–0.8) vs 0.7 (95% CI: 0.6–0.8), *p* = 0.698). Likewise, Ozkaya et al., found a non-significant difference in SpA RI between women with adverse pregnancy outcome compared to women with normal outcome (0.60 ± 0.37 vs 0.54 ± 0.10). Makikallio et al. described SpA RI during 6, 8, 9 and 11 weeks of gestation in 10 women having PE or preterm labor and compared them with 31 control pregnancies (week 6: 0.82 ± 0.06 vs 0.83 ± 0.06, week 8: 0.84 ± 0.04 vs 0.86 ± 0.06, week 9: 0.85 ± 0.04 vs 0.83 ± 0.06, week 11: 0.75 ± 0.09 vs 0.73 ± 0.09). During the whole study period, no significant differences between both groups were observed. Equally, our pooled meta-analysis data showed no significant differences in RI between complicated and healthy gestation throughout the first trimester (*p* = 0.568, data not shown). No data was available on RI measurements in second or third trimester pregnancies.

#### Spiral arteries located in the central part of the placental bed, versus those located at its periphery

Differences between central and peripheral SpA Doppler indices during the second trimester were investigated in two studies [9, 23]. Significantly higher PI and RI were found by Matijevic et al. in the SpA located at the peripheral parts of the placenta compared to the central SpA (peripheral SpA PI: 0.48 ± 0.28 (mean ± SD) vs central SpA 0.38 ± 0.26, *p* < 0.001; peripheral SpA RI: 0.38 ± 0.21 vs central SpA 0.32 ± 0.18, *p* < 0.001). No significant differences were found in the PSV between the central and peripheral spiral arteries (*p* > 0.05). Studies of Hsieh et al. concluded that PI and RI values of central placenta bed SpA seemed to be lower, although this finding was statistically non-significant due to the small number of subjects in their sample.

**Fig. 9:**
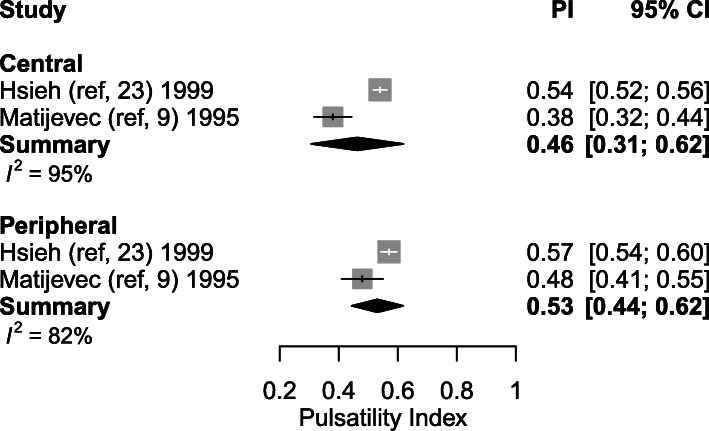
Forest plot of 2 studies exploring both central and peripheral spiral artery measurements during second trimester (Pulsatility Index)

**Fig. 10:**
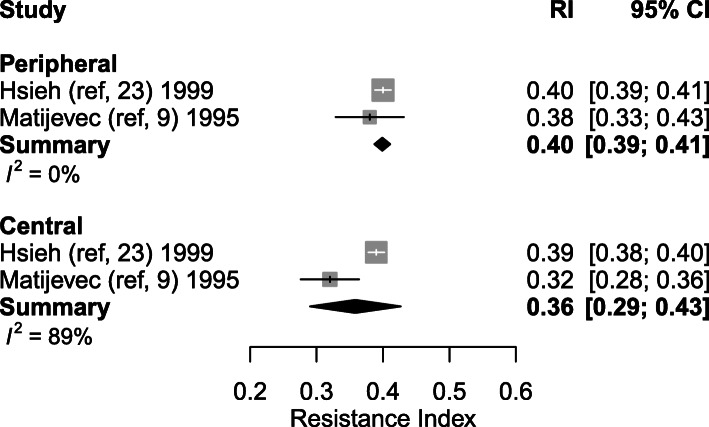
Forest plot of 2 studies exploring both central and peripheral spiral artery measurements during second trimester (Resistance Index)

Pooling these results, weighted mean PI in central SpA was 0.46 (95% CI: 0.31–0.62), compared to 0.53 (95% CI: 0.53–0.62) in peripheral SpA (*p* = 0.349) (Fig. 9). The weighted mean RI in central SpA was 0.36 (95% CI: 0.29–0.43) and 0.40 (95% CI: 0.39–0.41) in peripheral SpA (*p* = 0.584) (Fig. 10).

## Discussion

In healthy gestation, we observed a consistent decrease of PI and RI in SpA from the first to the second trimester, after which no relevant changes towards the third trimester were seen. Transformation of adrenergic-sensitive high-resistance into adrenergic-insensitive low-resistance SpA ensures unrestricted blood flow into the placenta [34, 35]. During early gestation, interstitial and endovascular trophoblast cells invade into SpA, where they start to replace the muscular wall and endothelium of these feeding vessels. Consequently, diameters of SpA broaden and lose their ability to respond to vasoactive stimuli, enabling continuous increased low-velocity low-resistance blood flow into the placental intervillous space and avoiding damage to placental tissue [1, 3, 6]. In parallel, total peripheral vascular resistance decreases and maternal cardiac output rises [36].

The exact time at which high oxidative maternal blood starts to flow into the intervillous space ranges from 5 to 12 weeks of gestation and is prevented earlier by the formation of endovascular trophoblast ‘plugs’ in the distal segment of the SpA [20, 21, 24]. These trophoblast plugs create a hypoxic placental environment that protects the foetus from oxidative stress and its damage [37]. The controversial data about timing of onset of maternal blood flow into the placenta could possibly be explained by the limited ultra-sonographic possibilities when attempting to visualize small vessels with low velocity. Roberts et al. [38] circumvented this limitation by using a contrast agent that enabled them to apprehend the vascular filling of the placenta. Although maternal blood flow into the placenta was detected at already 6 weeks of gestation, they concluded that microvascular flux into the intervillous space did not progressively increase until 13 weeks. After 13 weeks of gestation, significant changes in the nature of SpA blood flow parameters occur, as is illustrated by our results.

Defective remodeling of SpA can lead to a premature onset of maternal, high-flow and -oxidative blood into the placenta, generating mechanical and biochemical trophoblastic damage and increased apoptosis [39]. In PS-pregnancies, when oxidative stress is not yet sufficient to influence the preservation of the conceptus, transformation of the SpA walls will occur partially in the center of the placental bed and is limited to the decidual segments of the vessels. This results in vessels with high resistance, diminished perfusion and as a possible result the development of clinical hypertensive disorders or FGR [2, 6, 7].

Three of the included studies reported PI/ RI in healthy and complicated pregnancies [15, 27, 28]. The observed differences are based on purely first trimester measurements, while the first to second trimester change in PI/RI as sign of adequate spiral artery remodeling may be the pivot between uncomplicated and complicated pregnancy. Additionally, not all reported complications are strictly associated with defective spiral artery remodeling, possibly confounding the results.

Pijnenborg et al. [40] found that with enlargement of the placental site, the SpA in the peripheral parts of the placenta come to lie more obliquely and causes their distal segments to lie more parallel to the basal plate. Multiple openings are formed in the walls of the SpA that are in connection with the intervillous space, depriving the more distal segments of SpA of blood flow and leading to local decidual necrosis in the peripheral parts of the placenta. Jauniaux and Burton put another theory forward that there may be a developmental gradient in the extent of extravillous trophoblast plugging, being the greatest in the central part of the placenta. The onset of the maternal flow into the placenta starts with unplugging of the SpA, which is more extensive in the peripheral parts and leads to a local hyperoxic environment. Higher levels of oxygen cause oxidative stress, induce a down-regulation of angiogenic growth factors and inhibit the invasive and proliferative activity of the trophoblast [41–43]. These changes in peripheral SpA could explain the previous described differences in Doppler measurements between central and peripheral parts in the placenta [9, 23] and a single placental (sono-)biopsy may not be representative for the entire vascular placental bed given the dissimilitude in impedance [44]. However, our meta-analysis could not confirm statistically significant differences between central and peripheral SpA measurements when pooling the data. Moreover, most of the included articles in our meta-analysis did not specify where in the placenta the measurement was made [15, 21, 25, 28] or only measured central SpA [2, 20, 22, 26, 27].

The use of Doppler ultrasonography offers a non-invasive technique for the measurement of SpA flow [9]. Nonetheless, results on Doppler velocimetry of SpA are controversial, and possibly explained by the bloom artefact where the Doppler signal diverges beyond the vessels walls [10, 45]. Measurement of SpA could be challenging due to their small diameter and torturous character, although modernistic improvement in ultrasound imaging and the validation of protocols may overcome this problem [15, 46, 47]. Inter-observer reproducibility of uterine measurements is strongly correlated with the experience of the ultra-sonographer, so presumptively this also applies for SpA [46]. Tekay et al. [48] found that the main sources of intra-observer variance in uterine measurements include maternal heart rate, breathing, blood pressure and placenta localization. None of our included studies showed results for these variables. All used colour Doppler, while the use of Power Doppler could increase the precision and its clinical appliance when measuring SpA [49].

Lost to follow up was not documented in the longitudinal designs, possibly influencing the constructed meta-regression curves. Most studies only performed measurements during first trimester, and those studies used transvaginal probes, whereas transabdominal probes were used in studies measuring during the second and third trimester. However, Marchi et al. [46] showed that measurements of the uterine arteries during first trimester can be performed with similar results in both transabdominal and transvaginal approach. Obesity may influence transabdominal examinations quality [15, 46]. Unfortunately, quality assessment revealed insufficient reporting of maternal weight or body mass index, which interferes with the interpretation of the measurements. Furthermore, no conformity of used ultrasound settings was observed between studies and publication bias could not be excluded given the small amount of studies that met the inclusion criteria in this review. Last, we only included studies that were written English language, which could possibly induce a source of bias. However, previous studies have concluded there is no evidence of a systematic bias from the use of language restriction in systematic review-based meta-analyses in conventional medicine [50–52]. As all authors must agree with the content of the paper, all authors must at least be able to judge the content of included studies for systematic reviewing. If we would not use language restriction, it would influence the applicability and reliability of our results. Besides, out of 93 articles that were not published in English language, only 5 articles (5%) were applicable for full text analysis based on title and abstract. Given these considerations, we only included papers written in a language that could be weighed on validity by all authors.

## Conclusions

Despite the fact that upstream uterine measurements are closely related to downstream SpA measurements [22], it is assumable that measuring resistance downstream could reflect the pathological process underlying placenta insufficiency related diseases in a superior practice. This review and meta-analysis concludes that an observed decrease of SpA PI and RI from the first towards the second trimester parallels the physiological trophoblast invasion converting SpA during early gestation. Higher PI was found in SpA of complicated pregnancies compared to healthy pregnancies, possibly reflecting suboptimal utero-placental circulation and the risk of maternal and foetal complications. Although individual studies have identified differences between SpA located in the central part of the placental bed versus those located at its periphery, this meta-analysis could not confirm this. Longitudinal studies examining comprehensively the predictive value of spiral artery Doppler for complicated pregnancies are yet to be carried out.

## Data Availability

The datasets generated and analysed during the current study are available from the corresponding author on reasonable request.

## Abbreviations

SpA: Spiral artery spiral arteries
PS: Placenta syndrome
PE: Preeclampsia
FGR: Foetal growth restriction
PI: Pulsatility index
RI: Resistance index
PSV: Peak systolic velocity
CI: Confidence interval
SD: Standard deviation
vs: Versus
e.g.: Exempli gratia = for example
